# Improving cardiometabolic health through nudging dietary behaviours and physical activity in low SES adults: design of the Supreme Nudge project

**DOI:** 10.1186/s12889-018-5839-1

**Published:** 2018-07-20

**Authors:** Jeroen Lakerveld, Joreintje D. Mackenbach, Femke de Boer, Boris Brandhorst, Jacqueline E. W. Broerse, Gert-Jan de Bruijn, Gerda Feunekes, Marleen Gillebaart, Marjolein Harbers, Jody Hoenink, Michel Klein, Frederike Mensink, Cédric Middel, Denise T. D. de Ridder, Femke Rutters, Ivonne Sluijs, Yvonne T. van der Schouw, Tjerk Jan Schuitmaker, Saskia J. te Velde, Elizabeth Velema, Wilma Waterlander, Johannes Brug, Joline W. J. Beulens

**Affiliations:** 1 Department of Epidemiology and Biostatistics, Amsterdam Public Health research institute, VU University Medical Center, Amsterdam, the Netherlands; 2 Department of Social, Health and Organizational Psychology, Utrecht University, Utrecht, the Netherlands; 3 University of Amsterdam, Amsterdam School of Communication Research ASCoR, Amsterdam, the Netherlands; 4 Athena Institute, Faculty of Science, VU University Amsterdam, Amsterdam, the Netherlands; 5 Netherlands Nutrition Centre, the Hague, the Netherlands; 6 Julius Center for Health Sciences and Primary Care, University Medical Center Utrecht, Utrecht, the Netherlands; 7 Department of Computer Science, VU University Amsterdam, Amsterdam, the Netherlands; 8 Te Velde Research & Consultancy, Bunnik, the Netherlands; 9 Department of Public Health, Amsterdam Public Health Research institute, Academic Medical Centre, University of Amsterdam, Amsterdam, the Netherlands

**Keywords:** Nudging, Supermarket, Pricing, Physical activity, Dietary behaviour, Low socio-economic status, Cardiometabolic health, M-health, Food environment

## Abstract

**Background:**

Initiating and maintaining a healthy lifestyle -including healthy eating and sufficient physical activity- is key for cardiometabolic health. A health-promoting environment can facilitate a healthy lifestyle, and may be especially helpful to reach individuals with a lower socio-economic status (SES). In the Supreme Nudge project, we will study the effects of pricing and nudging strategies in the supermarket – one of the most important point-of-choice settings for food choices – and of a context-specific mobile physical activity promotion app. This paper describes the stepwise and theory-based design of Supreme Nudge, which aims to develop, implement and evaluate environmental changes for a sustained impact on lifestyle behaviours and cardiometabolic health in low SES adults.

**Methods:**

Supreme Nudge uses a multi-disciplinary and mixed methods approach, integrating participatory action research, qualitative interviews, experimental pilot studies, and a randomized controlled trial in a real-life (supermarket) setting. First, we will identify the needs, characteristics and preferences of the target group as well as of the participating supermarket chain. Second, we will conduct a series of pilot studies to test novel, promising and feasible intervention components. Third, a final selection of intervention components will be implemented in a full-scale randomised controlled supermarket trial. Approximately 1000 low SES adults will be recruited across 8–12 supermarkets and randomised at supermarket level to receive 1) no intervention (control); 2) environmental nudges such as food product placement or promotion; 3) nudges and a tailored physical activity app that provides time- and context specific feedback; 4) pricing interventions, nudges, and the physical activity app. The effects on dietary behaviours and physical activity will be evaluated at 3, 6 and 12 months, and on cardiometabolic health at 6 and 12 months. Finally, we will evaluate the Reach, Effectiveness, Adoption, Implementation and Maintenance (RE-AIM) of the intervention, and we will use insights from System Innovation and Transition Management theories to define the best strategies for implementation and upscaling beyond the study period.

**Discussion:**

The Supreme Nudge project is likely to generate thorough evidence relevant for policy and practice on the effects of a mixed method and multi-disciplinary intervention targeting dietary behaviours and physical activity.

**Trial registration:**

The real-life trial has been registered on 30 May 2018, NTR7302.

## Background

Unhealthy dietary behaviours and lack of physical activity account for over half of the burden of type 2 diabetes and cardiovascular diseases (CVD), and both behaviours are important determinants of other major non-communicable diseases such as cancer and neurodegenerative diseases [1]. These unhealthy behaviours tend to cluster in people with a lower socio-economic status (SES) [2], making this an especially vulnerable group and a key focus for intervention approaches. Social cognitive theories suggest that individual-level factors such as attitude, knowledge and motivation are important determinants of lifestyle behaviours and should be intervened upon via educational strategies [3–6]. However, educational interventions targeting these individual-level level factors often lack sufficient effects in changing behaviours and health, especially on the longer term, and particularly in lower SES groups [7]. One reason for this may be the lack of sufficient consideration of the social, physical and economic context in which these health behaviours take place. Socio-ecological models posit [8, 9], and a growing body of evidence shows, that environmental interventions, i.e., changing food and/or physical activity environments, can be effective in changing dietary behaviours, physical activity and as a result cardiometabolic health [10–13]. As environmental interventions become incorporated into structures and systems [14, 15], they have the potential to sustainably make the healthy choice the easy choice. An additional benefit is that environmental changes reach all those who are exposed to these environments, including populations that are considered ‘difficult-to-reach’ in traditional experiments, such as people with a low SES [14, 16]. With the Supreme Nudge project we aim to develop, implement and evaluate environmental changes for a sustained impact on lifestyle behaviours and cardiometabolic health in low SES adults.

### Pricing, nudging and food choice

Pricing and nudging strategies are expected to be promising environmental strategies. Indeed, the first economic law of demand states that if pricing of a certain product increases, demand will decrease, and vice versa [17]. Pricing strategies such as subsidies or taxes are effective in changing food purchases and consumption [18], where greater effects may be accomplished by combining subsidies on healthier products and taxation of unhealthier products [19]. Discounts on fruits and vegetables increase the purchase and intake of fruit and vegetables [20–23], whereas recently implemented food taxes reduced purchases when prices were increased; especially among low-SES individuals [24, 25]. Vulnerable populations, including lower SES consumers, are most price-responsive and, in terms of health, may benefit most from changes in the relative prices of foods and beverages [19, 26, 27].

However, the dual process theory describes that although individuals make choices based on both their rational, reflective system, they also use their impulsive, automatic system [28]. Nudging - also referred to as ‘choice architecture’ - encompasses subtle environmental changes that are implemented to make the desired choice more likely, without eliminating or forbidding the alternative option or changing economic incentives [29]. Nudges specifically target the heuristic choices, rather than rational, reflective choice and as such do not require conscious decision making [28, 30, 31]. The contemporary scientific literature demonstrates that nudging has great potential to influence food purchase- and intake behaviours [32–35], as well as physical activity [36], and is accepted by the public [37–39].

Although the evidence base on pricing and nudging strategies is growing, evidence is largely restricted to short-term effects on food purchases and (proxies of) health behaviours. Little is known about the combined and long-term impact of such integrated interventions on behaviours and cardiometabolic risk factors, and how to best implement these strategies in real life policy and practice.

### Supermarket environment

Supermarkets play a central role in the food system. In large parts of the world, including the Netherlands, supermarkets are the place where people buy the majority of their food [40, 41]. Furthermore, generally a small number of companies hold a large share of the retail food market [42], implicating that changes by one or two retail chains have the potential to affect dietary choices of the population at large. As such, supermarkets provide a suitable setting for implementing and testing pricing and nudging strategies at the point of purchase, and can play a crucial role in developing strategies that can be adopted and sustained long-term.

### Physical activity

Point of dietary choice settings such as supermarkets are well-suited to promote more healthful diets, but improving cardiometabolic health also requires changes in physical activity levels. Social cognitive and self-regulation theories suggest, and research has shown, that it is more likely for physical activity interventions to be effective if approaches are individually tailored and provide context-specific feedback on physical activity opportunities and performance levels [36]. Indeed, individually tailored feedback, goal setting and advice on dietary and physical activity behaviours have been shown to be superior to generic and more traditional health education attempts [43]. By making use of artificial intelligence and present those via app-based mobile technology, individually tailored and context-specific feedback can be computed automatically and provided instantaneously. The GPS sensor in smartphones and geo-fencing techniques enables users to receive prompts at crucial as well as contextual decision-making moments, e.g., choosing between taking their bicycle or car, or between taking the stairs or the elevator. The fundaments for a Supreme Nudge app that will provide such feedback were recently developed in our lab, following a systematic process and using theory-based behaviour change techniques [44]. The model-based and adaptive predictions used in that app enable highly tailored feedback, i.e. not only tailored to the user’s physical and social context and beliefs, but also communicated in a way that fits the user’s preferences, which is critical for actually reaching the low-SES users and support them to make healthy physical activity choices. The integration of informatics, psychology, and communication and media studies in the development of an adaptive individual and context-tailored ‘mHealth’ (‘mobile health’) intervention has great potential to promote physical activity, especially in high risk groups such as low-SES adults [45].

### Implementation and upscaling

In order to induce sustainable behavioural changes and increase the maintenance of interventions all stakeholders need to be involved from the start of the project [46–48]. Community projects or multilevel interventions that have involved the target group from the start, taking a bottom-up approach, are generally more effective in reaching those most in need of the intervention. Co-creating intervention components is also more likely to generate the desired change, as the intervention components focus on behaviours or settings that are meaningful and feasible to the target group [49–54]. Involving stakeholders from food retail will provide insight into the dynamics of supermarkets operation and the barriers and opportunities to make the supermarket environment healthier, for instance through price deals or contracts with manufacturers and/or suppliers. Upscaling the intervention also requires the involvement of stakeholders, to embed the intervention components within a social systems’ culture and structure, as opposed to mere dissemination [55]. Current knowledge on how to effectively scale up health behaviour change interventions is scarce [56]. Within Supreme Nudge, we will use insights from the Transition Theory to optimise the upscaling of the intervention using a three-step approach: First, to gather evidence on the mechanisms and structures underlying effects (deepening). Second, to replicate the intervention in different settings (broadening). And third, to embed the intervention in broader societal structures such as policies of government and other organizations (embedding). Central to this approach is a System Innovation that takes into account: 1) the culture, containing the values and perspectives held by actors in the system, 2) the structure, consisting of the rules and organisational elements guiding and constraining system practices, and 3) the practice, representing actions relevant to the system, carried out by agents in the system [55].

The combination of using an environmental approach targeting the supermarket environment to stimulate healthier food purchases and using a personalized approach to support physical activity seems promising for the initiation of a healthy lifestyle and improving cardiometabolic health. Investigating the implementation and the evaluation of such a combined intervention within Supreme Nudge enables us to address a range of knowledge gaps that are highlighted in previous studies, including: 1) testing the effectiveness of a combination of promising intervention strategies as opposed to a single intervention; 2) pre-testing intervention components in controlled environments that mimic real-life situations as best as possible; 3) provision of evidence on the longer-term effects, including cardiometabolic health indicators; and 4) insights into whether pricing and nudging strategies in the supermarket can be effectively used to facilitate a healthy diet.

The Supreme Nudge project has been designed to address these gaps, with the overall objective to carefully develop, implement and evaluate –including evaluation of the development and implementation- a combination of pricing, nudging, and individualized feedback strategies in a point-of-choice intervention package for low-SES adults, in order to improve dietary behaviours and physical activity levels and, ultimately, cardiometabolic health.

## Methods/design

The Supreme Nudge project comprises various qualitative and quantitative pilot studies, feeding into a real-life, cross-national intervention at the supermarket level. The latter study aims to improve cardiometabolic health through increasing healthier food purchases and intake through pricing and nudging, and through increasing physical activity through app-based tailored feedback and support. Coop – one of the major supermarket chains in the Netherlands – has formally agreed to participate in this project. Coop will provide support with the development of the environmental changes in the supermarket and will facilitate implementation of the intervention for the trial to evaluate the effects.

The following stepwise approach will be followed:

1. To identify the most promising food pricing, environmental nudging, and tailored physical activity feedback intervention components to change lifestyle behaviours according to characteristics and preferences of the target group and the supermarkets;
2. To pilot-test and adapt the range of intervention components;
3. To implement a multi-level intervention in a real-life setting using ‘pricing’, ‘nudging’ and ‘tailored feedback and support for physical activity’ and evaluate the effectiveness with regard to lifestyle behaviours and parameters of cardiometabolic health in a randomised controlled trial; and
4. To provide a roadmap to scale-up the intervention, including the identification of actors and factors that facilitate horizontal and vertical upscaling.

These steps are further detailed below.

### Identification of promising intervention components

#### Needs, characteristics and preferences of the target population

We will identify what food groups related to cardiometabolic health are most suitable for a nudging and pricing approach and how SES, sex, BMI and other potential moderating variables may influence these relations. This information will be derived by systematically reviewing and meta-analysing the existing evidence base, and through secondary quantitative analyses in large studies with comparable populations (such as the New Hoorn Study [57] SPOTLIGHT [58, 59] and EPIC-NL [60]). Second, Participatory Action Research (PAR) principles will be used to identify the needs of the target group as well as preferences towards environmental nudges and the content of a physical activity mobile telephone app.

#### Needs, characteristics and preferences of the supermarket stakeholders

A multidisciplinary reflexive assessment will be conducted to gather information from stakeholders in the Coop supermarket organisation on topics such as: the extent to which an unhealthy lifestyle, as a risk factor to cardiometabolic health, presents a societal problem; the responsibility of the supermarket and other food retail actors in promoting a healthier lifestyle; strategies to promote a healthier lifestyle and the embedding of such efforts in a supermarket; and the potential barriers and opportunities for this embedding. We will conduct qualitative interviews and focus groups among actors from all levels in the involved supermarket chain, to identify a range of perspectives. Subsequently, a questionnaire will be issued among Coop personnel at various levels, to develop a quantitative ranking of the fit of the identified perspectives with the overall organisation. The outcomes of the interviews and questionnaire will be validated and discussed in more depth with representatives of the interviewed stakeholder groups during an interactive workshop. In this workshop we will utilise methods such as actor and relationship mapping, root cause analysis, and preference ranking (for the comparison of options).

### Pilot-test and adapt the intervention components

Input from the target population and supermarket stakeholders will be taken into consideration when shortlisting the intervention components and their design. A previously used virtual supermarket environment will be taken as basis for a pilot test of the shortlisted intervention components in a screen-based virtual supermarket environment [23, 61]. Pilot tests will also be done in newly built 3D Virtual Reality (VR) supermarket. This may include the use of 3D VR devices such as the HTC Vive head-mounted displays. Building on feedback from the target group and progressive insights, an existing app to promote engaging in physical activities [44] will be enhanced and extended. This app was originally developed for a different target population than the current target population, i.e., young and higher educated adults. Therefore, the content of the feedback and motivational messages and the presentation of monitoring and social comparison results need to be adapted to media preferences and literacy levels of low-SES participants. Furthermore, new technological features will be pretested and if evaluated as being promising, they will be implemented in the app. Examples include geo-fencing and potentially socio-fencing techniques to enable context specific physical activity messages. Moreover, the original app was developed in combination with a *Fitbit One* activity tracker. However, it may be more feasible and appropriate to use the built-in accelerometers of smartphones for this target group. This requires additional testing, validating and adapting equations that translate the accelerations into physical activity data.

#### Testing of pricing and nudging supermarket intervention components?

We will examine the efficacy of three different types of nudging (‘social proof nudges’, ‘default nudges’ and ‘salient nudges’) that have previously shown robust effects [32, 62, 63] and might therefore also endure in a noisy and complex environment such as the supermarket. Social proof nudges make healthy food choice easier by the presenting information about other peoples’ food choices such as “Most sold in this supermarket” [64]. Default nudges change dietary choice by preselecting an option; for example having whole wheat bread as the standard option in the bread department, with white bread positioned more to the back [65, 66]. Salient nudges increase the visibility of the nudged food products and might include altering the placement of food items or using in-store banners to capture attention [67]. We will first explore the potential and working mechanisms of these three types of nudging in a series of studies using online and lab settings, during which decision-making styles, attention and situational factors will also be considered. The most promising of these nudges will then be piloted in a controlled virtual supermarket setting. Using a mixed design, a sample of ≈300 high and low SES participants will be recruited to perform five shops in a desktop-based virtual supermarket. With different versions of the virtual supermarket (i.e. the experimental conditions) we will tease out the effects of nudges, the effect of taxes and subsidies, the additional effects of tax/subsidy salience, and the combined effects of nudges, taxes, subsidies and tax/subsidy salience.

#### Testing of an intelligent and individually tailored physical activity intervention

We will perform a content-valuation and feasibility study with participants (*n* = 10) thinking out loud, while using the tailored physical activity app. Next, a small test panel will test the app during 8 weeks while a set of features of the app (i.e., geo-fencing, self-monitoring, social comparison, goal setting) will be switched on/off every week. Finally, qualitative interviews with the test panel will be held about the user experience, and preferences for app features will be assessed. Transcripts of interviews will be coded and organized in themes and analysed according to appropriate qualitative methods. Registered data on app usage will be analysed to see which functions and features are preferred and most valid across activity trackers. Analyses will account for differences in use and preferences between groups based on sex, BMI or physical activity level.

#### Interim evaluation and adaptation of intervention components

The intervention components will be adapted based on the studies as described above, and made suitable for implementation in a real-life setting. A workshop will be organized with representatives of the target group and supermarkets to formulate an evaluation framework, including the selection of process and outcome indicators and methods of data collection. Data collection for this interim evaluation will take place using a mix of observations, questionnaires, interviews and focus group discussions.

The aforementioned activities provide input for the larger, nation-wide randomised trial at the supermarket level. Key characteristics of this trial are described in detail below, and will be registered in the Netherlands Trial Registry.

### Implement and evaluate a multi-level intervention in a real-life setting

#### Intervention components, study setting & design

The study will be performed according to a randomized, controlled, parallel design. We will select eight-to-twelve supermarkets in closed market areas and low-SES communities in the Netherlands. Randomization will take place at the supermarket level. Included supermarkets will equally be randomised to one of the intervention arms, so that the recruited participants will be placed in one of the four groups:

1. Control group;
2. Intervention group 1: receiving ‘supermarket nudges’ only;
3. Intervention group 2: receiving ‘supermarket nudges’ and the physical activity app;
4. Intervention group 3: receiving ‘supermarket nudges’, the physical activity app and ‘supermarket pricing strategies’.

#### Participant recruitment

**Fig. 1:**
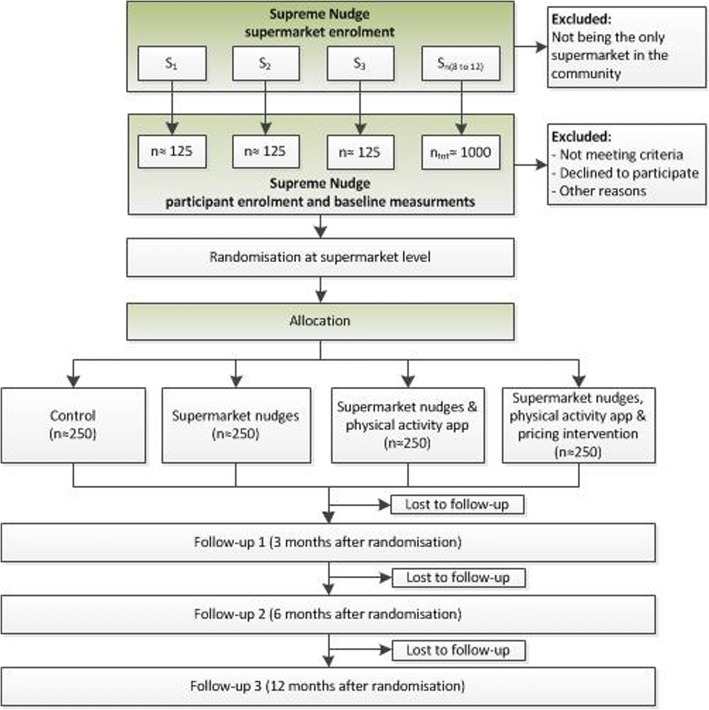
The Supreme Nudge randomised controlled trial profile

Study participants will be recruited at the included supermarket sites. Figure 1 summarises the recruitment strategy/sampling approach. Participants must provide written informed consent and comply with all of the following at inclusion: i) Age ≥ 18 years, ii) Having a low SES, based on educational attainment and/or income, and iii) Do their grocery shopping regularly at one of the selected supermarkets. The following exclusion criteria are defined: i) People who are unable to climb a flight of stairs or who have a contra-indication to engage in physical exercise, ii) Not being able to communicate adequately in the Dutch language, and iii) Not being regularly in the neighbourhood during the study period or moving house soon.

#### Measures

The primary outcomes of the trial include changes in parameters of metabolic health, namely: blood pressure, cholesterol values, HbA1c or glucose, and waist circumference at 6 and 12 months. Secondary outcomes are changes in objectively measured physical activity, dietary intake, food purchasing behaviour, food purchase intentions, customer satisfaction, type of purchase (e.g., habitual, impulsive, etc.), frequency of use of the mobile physical activity app, social cognitive factors (e.g., intention, awareness, self-efficacy, etc.); and implementation-related outcomes such as needs, characteristics and preferences of supermarkets and target populations, feasibility of the intervention and possibilities for scaling up, and the Reach, Adoption, Implementation and Maintenance (RE-AIM) of the intervention.

#### Timeline

The intervention phase will be 12 consecutive months to account for seasonal variation in shopping and physical activity behaviour and allow measurement of long-term effects. The short-term (3 months) and intermediate follow-up (6 months) will be used to measure changes in behaviours and intermediate psychological constructs, whereas the longer term follow up (12 months) will be used to evaluate changes in cardiometabolic health, and for the process-evaluation.

### Intervention embedding

#### Analysis of current culture, structure, and practice

Parallel to the intervention component design, an analysis will be performed of the Coop supermarket chain and its overarching system, to identify systemic leverage points and barriers for the successful cross-national embedding of the intervention. The analysis will follow the Unravelling Persistent Problems approach, which combines a structured literature study that unravels underlying structures with a focus on actor perspectives and relations [68]. First, a historical analysis of the Dutch food system is performed, to identify systemic mechanisms that impede or support embedding. Second, semi-structured interviews are conducted with stakeholders internal and external to the intervention, to explore their underlying mental structures, and resulting practices and perceptions of systemic leverage points and barriers.

#### Monitoring in real-life setting

During the real-life supermarket trial, reflexive monitoring sessions will be organized with relevant stakeholders (policy makers, researchers, Coop actors, target group), to reflexively evaluate the intervention, based on a co-created realist-evaluation framework, and in relation to the embedding process. Between sessions, newly developed strategies will be implemented by the relevant actors.

#### Developing a roadmap for embedding

From the evaluation outcomes, an overview will be developed of barriers encountered in practice, and of potential opportunities and strategies for intervention implementation and broader embedding. A strategic roadmap for intervention embedding will be co-created with the relevant stakeholder groups, based on interviews and co-creating workshops. These workshops facilitate the identification of visions and necessary steps to reach these end-goals, converging into a strategic roadmap for intervention embedding [69].

## Discussion

This paper describes the background and design of the Supreme Nudge project, a novel study that develops, implements and evaluates a combined intervention that includes environmental changes as well as tailored feedback to sustainably improve lifestyle behaviours and cardiometabolic health in Dutch low-SES adults. In the study, both initiation and maintenance of health-related behaviours will be targeted via distinct but connected procedures. Based on economic, psychological and public health theories, we hypothesize that changing the context in which individuals make lifestyle choices has a sustained impact on cardiometabolic health.

Such a complex, multi-disciplinary project brings about a number of challenges. The first challenge is the reliance on the willingness and commitment of participants, particularly of a low SES group that is difficult to reach. However, this project will take a bottom-up approach by first conducting a needs and preference assessment among the target group, and co-creating the intervention components. Also, participants to the real-life supermarket trial will be recruited directly from supermarkets located in low-SES areas via the opportunity for a non-invasive medical check-up. Moreover, the intervention approach, focusing mostly on the context in which behavioural choices are made, is tailored to work across all SES groups. Although individual-level interventions generally show limited effect among such groups, previous research showed that tailored feedback is more appreciated among lower educated respondents than among the higher educated [70, 71]. The second challenge is the collaboration with a supermarket chain and conducting a study in a real-life setting. The design and implementation of the intervention components will be dependent on the commitment and resources in the supermarkets. To limit corresponding risks, we involved the Coop supermarket chain and committed them to collaborate from the start to contribute to and support the project. In order to ensure that participants are ‘exposed’ to the supermarkets under study, the trial will be implemented in municipalities with a closed market area, such that there are no other supermarkets around. In addition, we will select participants based on mainly shopping at the intervention supermarket and monitor purchases at other shops during the trial to account for such contamination.

The project benefits from a number of strengths. A major strength is the mixed method and inter-disciplinary approach in which stakeholders are involved from the start. In addition, the project will start with a series of dynamic and smaller-scale experiments of which the results will gradually feed into the set-up of a large real-life randomised controlled trial. Objective measures are used for the primary outcomes, and for measuring physical activity. The latter is important as self-reported measures have been shown to be less valid, especially in low SES populations [72]. Due to the co-designed approaches and the committed involvement of one of the largest Dutch supermarket chains (Coop), the results are likely to have high applicability and external validity for participants and decision makers [73].

This five-year project started in July 2017 and is likely to generate unique insights into the short-term and long-term effects of interventions that use (a combination of) pricing, nudging, and tailored feedback to change behaviours and cardiometabolic health. The results may be used to identify entry-points for population-based approaches – with a specific focus on low SES individuals.

## Abbreviations

BMI: Body mass index
EPIC-NL: Dutch Contribution to European Prospective Investigation into Cancer and Nutrition
HbA1c: Glycosylated Haemoglobin, Type A1C
KORA: Cooperative Health Research in the Region Augsburg
mHealth: Mobile health
MPDS: Mass-production and delivery system
PAR: Participatory action research
RE-AIM: Reach, effectiveness, adoption, implementation, maintenance
SES: Socio-economic status
SPOTLIGHT: Sustainable prevention of obesity through integrated strategies
VR: Virtual reality
